# Pharmacological studies of the mechanism and function of interleukin-1β-induced miRNA-146a expression in primary human airway smooth muscle

**DOI:** 10.1186/1465-9921-11-68

**Published:** 2010-06-02

**Authors:** Hanna M Larner-Svensson, Andrew E Williams, Eleni Tsitsiou, Mark M Perry, Xiaoying Jiang, Kian F Chung, Mark A Lindsay

**Affiliations:** 1 Airway Disease, National Heart and Lung Institute, Imperial College London SW3 6LY, UK; 2 NIHR Translational Research Facility in Respiratory Medicine, University of Manchester, Education and Research Centre, Wythenshawe Hospital, Southmoor Road, Manchester M23 9LY, UK

## Abstract

**Background:**

Despite the widespread induction of miR-146a during the innate immune response little is known regarding its biogenesis, function and mechanism. We have therefore examined the role of miR-146a during the interleukin (IL)-1β-stimulated IL-6 and IL-8 release and proliferation in primary human airway smooth muscle (HASM) cells.

**Methods:**

HASM cells were isolated from human lung re-section, cultured to a maximum of 3 - 6 passages and then exposed to IL-1β. miR-146a expression were determined by qRT-PCR, IL-6 and IL-8 release by ELISA and proliferation using bromodeoxyuridine incorporation. The role of NF-κB and the MAP kinase pathways was assessed using pharmacological inhibitors of IKK2 (TPCA-1), JNK (SP600125), p38 MAP kinase (SB203580) and MEK-1/2 (PD98059). miR-146a function was determined following transfection of HASM with inhibitors and mimics using Amaxa electroporation.

**Results:**

IL-1β induced a time-dependent and prolonged 100-fold induction in miR-146a expression, which correlated with release of IL-6 and IL-8. Exposure to IL-1β had no effect upon HASM proliferation. Pharmacological studies showed that expression of primary miR-146a was regulated at the transcriptional levels by NF-κB whilst post-transcriptional processing to mature miR-146a was regulated by MEK-1/2 and JNK-1/2. Functional studies indicated that IL-1β-induced miR-146a expression does not negatively regulate IL-6 and IL-8 release or basal proliferation. However, inhibition of IL-1β-induced IL-6 and IL-8 release was observed at the super-maximal intracellular miR-146a levels obtained by transfection with miR-146a mimics and indicates that studies using miRNA mimics can produce false positive results. Mechanistic studies showed that in the presence of super-maximal levels, the action of miR-146a mimics was mediated at a step following IL-6 and IL-8 mRNA transcription and not through down-regulation of IL-1 receptor associated kinase 1 (IRAK-1) and TNF receptor-associated factor 6 (TRAF6) protein expression, two predicted miR-146a targets involved in IL-1β signalling.

**Conclusions:**

We have shown that IL-1β-induced miR-146a expression in HASM and that this was regulated at the transcriptional level by NF-κB and at the post-transcriptional level by the MEK-1/2 and JNK-1/2. Unlike previous reports, studies using miRNA inhibitors showed that miR-146a expression did not regulate IL-6 and IL-8 release or proliferation and suggest miR-146a function and mechanism is cell-type dependent.

## Introduction

Human airway smooth muscle (HASM) cells regulate both the tone and diameter of the respiratory airways. Inappropriate contraction of HASM in response to environmental stimuli is responsible for the reversible airways contraction that is associated with asthma, a chronic disease that affects approximately 10% of children and 5% of adults in Western countries [1,2]. In addition to their role in constriction, HASM cells are also thought to contribute towards the chronic inflammation and airway re-modelling that is characteristic of asthma [3,4]. Thus, HASM cells have been shown to release a host of inflammatory mediators such as IL-6, IL-8, eotaxin, matrix metalloproteinase-12 and prostaglandin E_2 _and to undergo proliferation in response to activation via the Toll like receptor (TLR)/interleukin (IL)-1 receptor family [5-13].

Members of the TLR/IL-1 receptor family possess a common intracellular domain and can be subdivided into the TLR family that comprises at least 11 members and the IL-1R family that has 10 members [14,15]. The TLRs recognise conserved molecules derived from bacteria, fungi and viruses and contribute towards the innate immune response whilst the IL-1Rs are activated by the pro-inflammatory cytokines, IL-1α, IL-1β, IL-18 and IL-33 [15]. Agonism of these receptors leads to the activation of a common intracellular signalling pathway. The initial step involves association with the adaptor protein myeloid differentiation primary-response gene 88 (MyD88), which recruits IL-1R associated kinase 1 (IRAK-1) and TNF receptor-associated factor 6 (TRAF6). In HASM cells, these receptors activate a variety of intracellular signalling pathways and pro-inflammatory transcription factors. One of the most important is NF-κB, which under basal conditions is localized within the cytoplasm bound to IκBα. Degradation of IκBα following phosphorylation by I-κB kinase-2 (IKK-2) results in the nuclear translocation of activated NF-κB, DNA binding and subsequent transcription of multiple inflammatory mediators [6,16,17]. Alternative pathways that are known to be activated in HASM cells include the mitogen activated kinase cascades that terminate at ERK-1/2, JNK-1/2 and p38 MAP kinase [6,10,17-21].

miRNA-mediated RNA interference has been identified as a novel mechanism that regulates gene expression at the translational level [22,23]. These short RNA sequences of 20-23 nucleotides are produced by the processing of full length mRNA-like transcripts known as primary miRNAs [24,25]. These larger primary miRNA transcripts undergo enzymatic cleavage by the RNAse III enzyme Drosha to produce ~70 nt precursor miRNAs. These are then transported to the cytoplasm where they are further processed by another RNAse III enzyme, DICER, to produce ~21-23 double stranded RNA. One strand, the mature miRNA, is then loaded into the RNA-induced silencing complex (RISC) where it is believed to either repress mRNA translation or reduce mRNA stability following imperfect binding between the miRNA and the miRNA-recognition elements (MRE) within the 3' untranslated region (UTR) of target genes. Specificity of the miRNA is thought to be primarily mediated by the 'seed' region that is localised between residues 2-8 at the 5' end [26-28]. Interestingly, recent studies have identified miRNA-mediated RNA interference as a potentially novel mechanism that regulates the immune response [29-31]. In particular, rapid increases in miR-146a and miR-155 expression have been demonstrated in immune cells following activation of members of the TLR/IL-1R family [32-37]. Since these initial observations, miR-155 has been shown to regulate multiple responses associated with the innate and acquired immune response including LPS-induced release of inflammatory mediators from monocytes, T cells and B cells [34,38,39], proliferation and differentiation of myeloid and lymphoid cells [40-44] and B cell antibody switching [45-47]. Significantly, these studies indicate that the function and mechanism of miR-155 is dependent upon the cell-type and stage of development/differentiation.

In contrast to miR-155, much less is known regarding the biological role of miRNA-146a. This is despite its widespread induction in both immune and structural cells, such as alveolar and airway epithelial cells, monocytes/macrophages, fibroblasts and chondrocytes following the initiation of the innate immune response [32,48-54]. Studies into the mechanisms that regulate miR-146a expression has demonstrated that the initial transcription of primary miR-146a is mediated via activation of NF-κB [32,48,49,55,56]. In contrast, nothing is known regarding the mechanisms that regulate the processing of primary miR-146a to produce the mature miR-146a. Interestingly, recent studies have indicated that TGFβ-induced miR-21 production in human pulmonary artery smooth muscle is primarily regulated at the level of Drosha, which processes primary miR-21 to precursor miR-21, and is driven by activation of the Smad signalling pathway [57]. Evidence of the importance of post-transcriptional regulation has also been provided from studies of the single-strand RNA-binding protein KH-type splicing regulatory protein (KSRP). This has been shown to serve as a component of both Drosha and Dicer complexes and regulates the biogenesis of a subset of miRNAs through binding with high affinity to the terminal loop of the target miRNA precursors and promoting their maturation [58]. In particular, KSRP has been shown to regulate the maturation miR-155 and the subsequent down-regulation of inflammatory mediators following LPS-stimulation of bone marrow derived macrophages [39].

Functional studies indicate that miR-146a negatively regulates the release of inflammatory mediators, although there are differing reports as to the precise mechanism of action [32,48,49]. Taganov *et al *[32] have suggested that miR-146a targets the down-regulation of IRAK-1 and TRAF6, which are located in the TLR/IL-1R signalling pathway. This hypothesis has been supported by recent studies of miR-146a mediated down-regulation of IFN-β release in vesicular stomatitis virus (VSV)-infected mouse peritoneal macrophages [49]. In contrast, our previous studies in IL-1β-stimulated human alveolar A549 epithelial cells indicated that miR-146a attenuated IL-8 and RANTES release at a step following their transcription and not through the targeting of IRAK1 and TRAF6 [48].

To further characterise the function and mechanism of action of miR-146a, we have examined the IL-1β-induced response in primary HASM cells. In contrast to the rapid induction in miR-146a expression previously described [32,48,49], we observed a slow-developing and prolonged induction of miR-146a expression. We have confirmed that NF-κB regulates miR-146a transcription and demonstrate for the first time, that the post-transcriptional processing of primary miR-146a to mature miR-146a is regulated by MEK-1/2 and JNK-1/2. Significantly, functional studies indicated that IL-1β-induced miR-146a expression is not central to the negative regulation of IL-6 and IL-8 release or basal proliferation in HASM cells under physiological conditions. However, we demonstrated that transfection with super-maximal levels of miR-146a could inhibit IL-1β-induced IL-6 and IL-8 release and under these conditions, we confirmed our previous observation that the action of miR-146a was mediated at a step following the transcription of IL-6 and IL-8 and not through down-regulation of IRAK-1 and TRAF6.

## Methods

### Ethics Statement

This study received written approval from the National Heart and Lung Institute and Royal Brompton Hospital NHS Trust Ethics Committee and all subjects gave informed written consent to participate in the study.

### Isolation and culture of human airway smooth muscle cells

HASM was obtained from lobar or main bronchus of patients undergoing lung resection for carcinoma of the bronchus. The smooth muscle was dissected out under sterile conditions and placed in culture. Cells were maintained in Dulbecco's modified Eagle's medium (DMEM) containing 10% foetal calf serum (FCS) supplemented with sodium pyruvate (1 mM), L-glutamine (2 mM), penicillin (100 U/ml) streptomycin (100 μg/ml) and amphotericin B (1.5 μg/ml) in a humidified atmosphere at 37°C in air/CO2. HASM cells at passages 3-6 from 20 different donors were used in the studies described.

### Cell stimulation

HASM cells were plated onto 6-well plates for assessment of cytokine release and RNA extraction. Prior to experiments, confluent cells were growth-arrested by FCS deprivation for 24 h in DMEM supplemented with sodium pyruvate (1 mM), L-glutamine (2 mM), nonessential amino acids (1:100), penicillin (100 U/ml)/streptomycin (100 μg/ml), amphotericin B (1.5 μg/ml), and bovine serum albumin (0.1%). Cells were stimulated in triplicate in a fresh FCS-free medium with the indicated IL-1β concentration or with 1 ng/ml IL-1β for indicated times. To examine the effect of the inhibitors of JNK (SP600125) [54,55], IKK2 (TPCA-1) [56,57], p38 MAP kinase (SB203580) [58,59] and MEK-1/2 (PD98059) [60,61] the indicated concentration (in the range 0.1 to 10 μM) was added 60 min prior to the addition of IL-1β (1 ng/ml). At the indicated times, the levels of IL-6 and IL-8 were determined by DuoSet ELISA (R&D Systems) and the remaining cells were extracted for RNA (using a miRVana extraction kit, Ambion).

### Measurement of cell number

After the supernatants were removed from the cells, 200 μl of MTT solution (3-(4,5-dimethylthiazol-2-yl)-2,5-diphenyltetrazolium bromide) was added and left to incubate for 30 min or until sufficient colour developed. Cells were washed and 200 μl of DMSO (Sigma-Aldrich) was added to each well. The optical density (OD) was measured at 550 nm using a spectrophotometer plate reader and expressed as a % of the control.

### Measurement of cell proliferation

Cell proliferation was quantified using a DNA bromodeoxyuridine (BrdU) incorporation assay (Roche Applied Science, Burgess Hill, UK). The amount of incorporated BrdU is a measure of the rate of DNA synthesis of the cells and thus indirectly of cell proliferation. The cell proliferation kit was used according to the manufacturer's instructions. Briefly, HASM cells were seeded in DMEM containing 10% FCS in 96-well cell culture plates at a density of 3,500 cells/well. At 30-50% confluence, the medium was changed to required concentration of FCS and cells were treated with/out IL-1β (1 ng/ml) for indicated time. At 24 h prior to the end of the stimulation period, BrdU labelling solution were added to each well at a final concentration of 10 μM. At the end of the stimulation period, cells were fixed (60 min) and then incubated for 90 min at room temperature, with 1/100 dilution of peroxidase labelled anti-BrdU antibody. The wells were then washed three times, incubated for 5 mins at room temperature with substrate solution and the luminescence was measured using a Fluorostar plate reader (BMG, Offenburg, Germany).

### Transfection with miR-146a mimics and inhibitors

HASM cells were transfected using Basic Nucleofector kit for primary smooth muscle cells according to manufacturer's instructions using Amaxa Nucleofector II device (program P-13) (Amaxa Biosystems, Cologne, Germany). miR-146a mimics and controls were obtained from Ambion/Applied Biosystems Ltd and locked nucleic acid (LNA)-based miR-146a inhibitors and controls were obtained from Exiqon Ltd. Transfected cells were plated into 6-well plates and left to adhere overnight before being serum starved for 6 h prior to stimulation with 1 ng/ml IL-1β. Supernatants were removed at 24 h and IL-6, IL-8 and IFN- levels were determined by DuoSet ELISA (R&D Systems). The remaining cells were extracted for RNA (using a miRVana extraction kit, Ambion) or tested for viability by MTT assay.

### Measurement of miRNAs, primary miR-146a and mRNA expression

Total RNA was extracted using the *mir*Vana™miRNA isolation kit (Ambion Europe) according to the manufacturer's instructions. RNA was eluted in 50 μl RNase-free water (Promega UK, Southampton, UK) and stored at -70°C. RNA content and purity was measured using a BioTek PowerWave XS (SSi Robotics, Tustin, CA, U.S.A.) spectrophotometer. miRNA expression profiling was carried out on total RNA extracts by two-step TaqMan^® ^reverse transcription polymerase chain reaction protocol (RT-PCR) as previously described [36]. mRNA expression levels of IRAK-1, TRAF6, IL-6 and IL-8 was determined using semi-quantitative two-step RT-PCR as previously described [62] using Assay on Demand primer/probe sets obtained from Applied Biosystems, UK. Primary miR-146a expression was determined using RT-PCR and Sybr green detection using the following primers: forward: CAAAGGCTTTGAAGGCCTCTCTA; reverse: ATGGCACCCAGCTGACCAT. All miRNA, primary miRNA and mRNA samples were normalised against 18 S (Applied Biosystems, Catalogue Number 4310893E). The separate well, 2-(ΔΔCt) method [63] was used to determine relative-quantitative levels of individual mRNAs, miRNAs and primary miR-146a, and these were expressed as the fold-difference to the relevant controls.

### Western Blotting

Proteins were extracted from HASM cells as previously described [59], separated upon 10% SDS-PAGE (Invitrogen) and transferred to nitrocellulose (Amersham Ltd). Protein (20 μg) were detected by Western blotting using a rabbit anti-TRAF6 antibody (H-274) [60], rabbit anti-IRAK-1 antibody (H-273)[61] obtained from Santa Cruz Biotechnology. All primary antibodies were used a concentration of 1:200 or 1:400 and were incubated overnight. Labelling of the first antibody was detected using relevant secondary antibodies conjugated to HRP (Dako Ltd) and detected using ECL reagents (Amersham Ltd., UK).

### Data and statistical analysis

The results presented are the mean ± SEM of at least three independent experiments. Statistical analysis was performed using the Mann-Whitney U-test which assumed non-parametric distribution. P values of < 0.05 were considered significant and are indicated with asterisks.

## Results

### IL-1β-induced a time- and concentration-dependent increase in miR-146a expression

**Figure 1:**
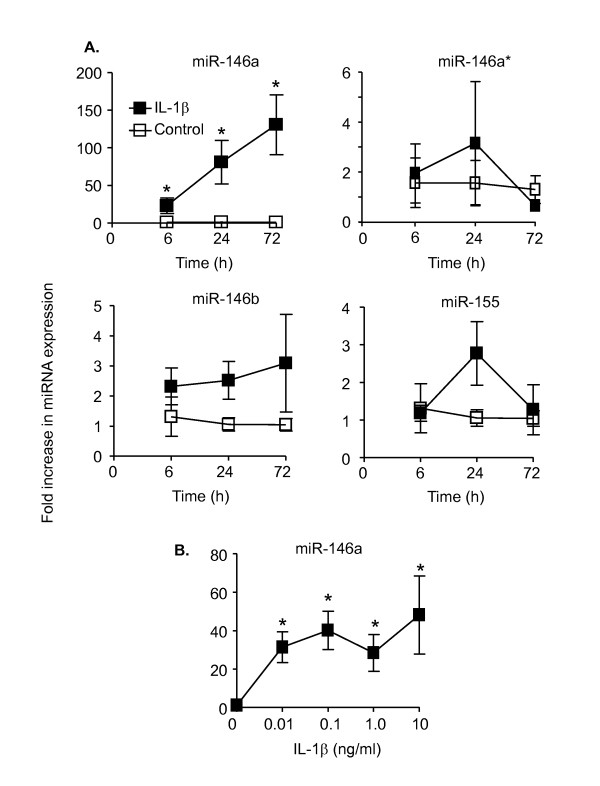
**IL-1β-induced a time- and concentration-dependent increase in miR-146a expression**. HASM cells were exposed to either vehicle control or 1 ng/ml IL-1β for the indicated time or to the indicated IL-1β concentration for 24 h. The time dependent expression of miR-146a, miR-146a*, miR-146b and miR-155 (A) or the concentration dependent miR-146a expression (B) was determined by qRT-PCR. The results are expressed as the mean ± SEM of four independent experiments where * p < 0.05 versus time-matched controls.

As previous investigations have implicated miR-146a and miR-155 in the regulation of TLR/IL-1R-induced response, we measured their expression following exposure to IL-1β (1 ng/ml) in HASM cells. Although there was variability between human donors, IL-1β caused a 23 ± 8-fold increase in miR-146a expression levels at 6 h, which continued to rise to 81 ± 29 and 131 ± 33-fold at 24 h and 72 h, respectively (Figure 1A). In contrast, we observed no significant changes in miR-146a* (reverse strand to the mature miR-146a strand), miR-146b or miR-155 levels (Figure 1A). Increasing IL-1β concentration showed that miR-146a expression was maximal at approximately 0.1 ng/ml (Figure 1B).

**Figure 2:**
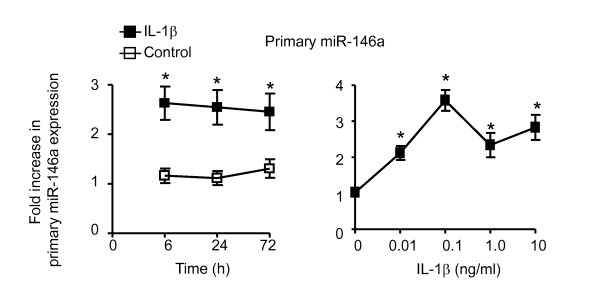
**IL-1β-induced a time- and concentration-dependent increase in primary miR-146a expression**. HASM cells were exposed to either vehicle control or 1 ng/ml IL-1β for the indicated time or to the indicated IL-1β concentration for 24 h. The time- (A) and concentration- (B) dependent expression of primary miR-146a was determined by qRT-PCR. The results are expressed as the mean ± SEM of four independent experiments where * p < 0.05 versus time-matched controls.

In subsequent studies, we measured the levels of the primary miR-146a in response to IL-1β. In contrast to mature miR-146a, primary miR-146a expression was increased by only 2-4 fold and maximal release was observed at 6 h, suggesting that the increase in mature miR-146a expression at 24 h and 72 h was due to regulation at the post-transcriptional level. Maximal expression of primary miR-146a production was observed at 0.1 ng/ml IL-1β (Figure 2).

### IL-1β-induced time- and concentration-dependent IL-6 and IL-8 release

**Figure 3:**
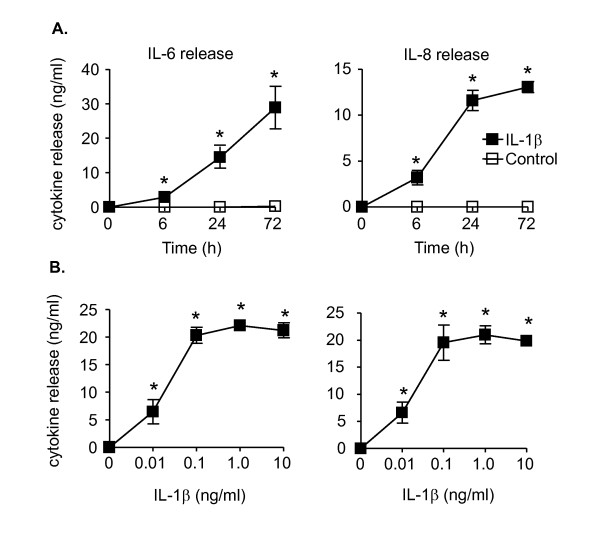
**IL-1β-induced a time- and concentration-dependent release of IL-6 and IL-8**. HASM cells were exposed to either vehicle control or 1 ng/ml IL-1β for the indicated time (A) or to the indicated IL-1β concentration for 24 h (B) before measurement of IL-6 and IL-8 release. The results are expressed as the mean ± SEM of four independent experiments where * p < 0.05 versus time-matched controls.

We subsequently assessed the effect of IL-1β upon the release of the pro-inflammatory mediators, IL-6 and IL-8 in HASM cells. IL-1β-induced a time- and concentration-dependent release of IL-6 and IL-8 (Figure 3). However, although we observed a significant elevation in both cytokines at 6 h, the IL-8 response reached a plateau at approximately 24 h, whilst IL-6 continued to increase throughout the 72 h period (Figure 3A). Examination of the effect of increasing IL-1β upon IL-6 and IL-8 release at 24 h showed similar concentration response curves with an EC_50 _value of ~0.03 ng/ml and maximal release at 1 ng/ml (Figure 3B). Given that we wanted to examine the role of miR-146a during IL-6 and IL-8 release subsequent studies were performed at 1 ng/ml IL-1β.

### IL-1β-induced miR-146a expression is regulated at the transcriptional and post-transcriptional level

In previous studies, we and others have demonstrated that IL-1β-induced activation of IKK2/NF-κB and the MAP kinases, ERK-1/2, JNK-1/2 and p38 MAP kinase in HASM cells and that these are inhibited in the presence of the selective pharmacological inhibitors of TPCA-1 (IKK2), PD098059 (MEK-1/2), SP600125 (JNK-1/2) and SB203580 (p38 MAP kinase), respectively [6,10,17-21]. We therefore used the biological active concentrations of these inhibitors to examine the role of the NF-κB and MAP kinases pathways during miR-146a expression.

**Figure 4:**
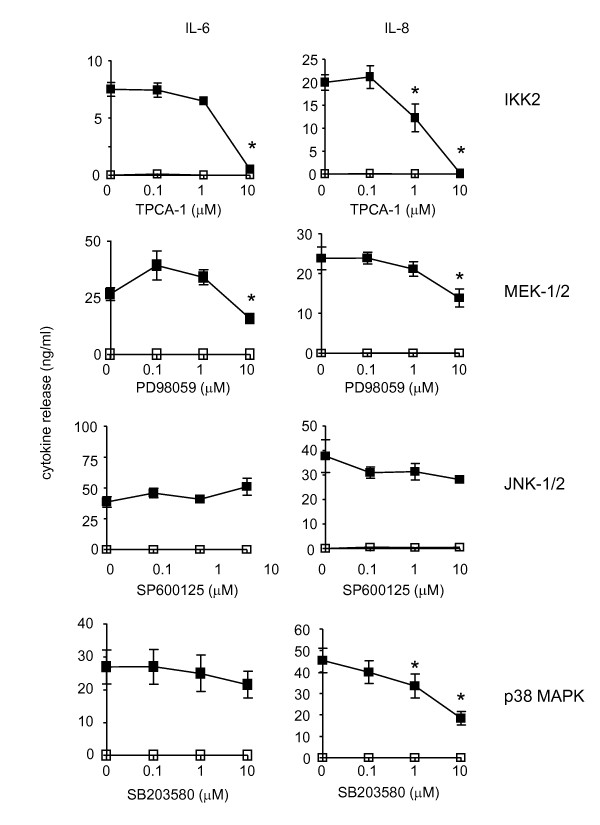
**Effect of inhibitors of IKK2 and MAP kinases upon IL-1β-induced IL-6 and IL-8 release**. HASM cells were pre-treated for 60 min with the indicated concentrations of the inhibitors of IKK-2 (TPCA-1), MEK-1/2 (PD098059), JNK-1/2 (SP600125) and p38 MAP kinase (SB203580). Following exposure to vehicle control or IL-1β (1 ng/ml) for 24 h the release of IL-6 and IL-8 was determined by ELISA. Results are the mean ± SEM of 3 independent experiments where * p < 0.05 versus IL-1β-stimulated cells.

**Figure 5:**
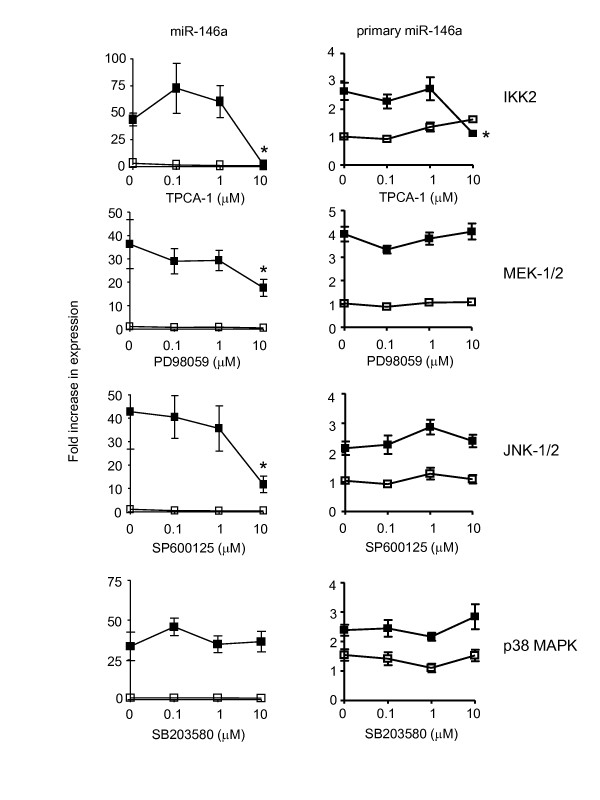
**Effect of inhibitors of IKK2 and MAP kinases upon IL-1β-induced miR-146a and primary miR-146a expression**. HASM cells were pre-treated for 60 min with the indicated concentrations of the inhibitors of IKK-2 (TPCA-1), MEK-1/2 (PD098059), JNK-1/2 (SP600125) and p38 MAP kinase (SB203580). Following exposure to vehicle control or IL-1β (1 ng/ml) for 24 h the expression of miR-146a and primary miR-146a was determined by qRT-PCR. Results are the mean ± SEM of 3 independent experiments where * p < 0.05 versus IL-1β-stimulated cells.

Following 60 min pre-treatment with inhibitors, HASM cells were stimulated with IL-1β and the generation of IL-6 (Figure 4), IL-8 (Figure 4), miR-146a (Figure 5) and primary miR-146a (Figure 5) were determined at 24 h. Exposure to TPCA-1 completely inhibited production of IL-6, IL-8 and miR-146a expression at 10 μM. This did not appear to have resulted from cell death since parallel studies showed a small (~15%) but non-significant reduction in cell viability (Additional File 1). The MEK-1/2 inhibitor (10 μM) also attenuated IL-6, IL-8 and miR-146a production although this was less pronounced than TPCA-1 inhibition and resulted in reductions of ~42%, 41% and 52%, respectively. In contrast, inhibition of the JNK-1/2 (SP600125) and p38 MAP kinase (SB203580) had differential actions upon cytokine and miR-146a production. Thus, JNK-1/2 inhibition had no effect upon IL-6 and IL-8 release but inhibited miR-146a expression, whilst blocking p38 MAP kinase inhibited IL-8 but not IL-6 or miR-146a production. In order to confirm these pharmacological studies, we also attempted to use siRNA-mediated knockdown to examine the role of IKK2 and the MAP kinases. Unfortunately, this was not possible since transfection with control siRNA blocked IL-1β-induced miR-146a expression (data not shown), possibly through competition between siRNA and primary/precursor miR-146a in the miRNA processing pathway.

Overall, pharmacological studies indicate that IL-1β-induced miR-146a expression is regulated via an IKK2-, MEK-1/2- and JNK-1/2-dependent pathway. Significantly, the effect of the JNK inhibitor indicated that IL-1β-induced miR-146a expression is not central to the regulation of IL-6 and IL-8 release. Thus, JNK inhibitor concentrations that attenuated mature miR-146a expression had no significant action upon IL-6 and IL-8 release.

To ascertain whether the actions of IKK2, MEK-1/2 and JNK-1/2 upon miR-146a expression were mediated at the transcriptional or post-transcriptional level, we also examined the action of these inhibitors upon expression of primary miR-146a (Figure 5). These investigations showed that primary miR-146a levels were attenuated by an inhibitor of IKK2 but not MEK-1/2 or JNK-1/2. Significantly, since these inhibitors were shown to have no effect upon cell viability (Additional File 1), this implied that miR-146a expression was regulated at the transcriptional level through activation of IKK2 (i.e. through NF-κB activity), whilst the post-transcriptional processing of primary miR-146a to produce mature miR-146a is regulated via a MEK-1/2- and JNK-1/2-dependent mechanism.

### IL-1β-induced miR-146a expression does not negatively regulate IL-6 and IL-8 release

**Figure 6:**
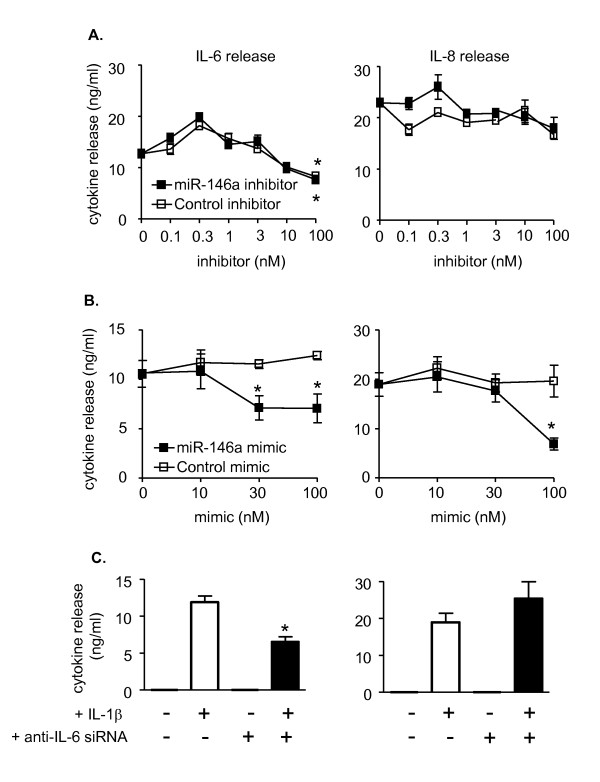
**Effect of miR-146a inhibitors and mimics upon IL-1β-induced IL-6 and IL-8 release**. HASM cells were electroporated in the presence of buffer (A-C), control inhibitor or miR-146a inhibitor (A), control mimic or miR-146a mimic (B) and control siRNA or an siRNA targeted at IL-6 (C). Cells were then exposed to vehicle control or 1 ng/ml IL-1β and the release of IL-6 and IL-8 was measured by ELISA at 24 h. The results are mean ± SEM of three independent experiments where * p < 0.05 versus IL-1β-stimulated cells.

In contrast to previous studies in alveolar epithelial cells and monocytes/macrophages, the studies using the JNK inhibitor suggested that increased miR-146a expression did not negatively regulate the release of inflammatory mediators. To clarify the role of miR-146a in the inflammatory response of HASM cells, we examined the action of miR-146a inhibitors and mimics on IL-1β-induced IL-6 and IL-8 release. In support of the observations using the JNK inhibitor, transfection using Amaxa electroporation showed that miR-146a inhibitors, at concentrations up to 100 nM, had no significant effect on IL-8 release (Figure 6A). In the case of IL-6, although the miR-146a inhibitor (100 nM) attenuated cytokine release this appeared to be a non-specific effect since this was also seen in the presence of the miRNA control inhibitor (Figure 6A). In contrast, the miR-146a mimic (100 nM) produced 23% and 62% reduction in IL-1β-induced IL-6 and IL-8 release, respectively (Figure 6B).

**Figure 7:**
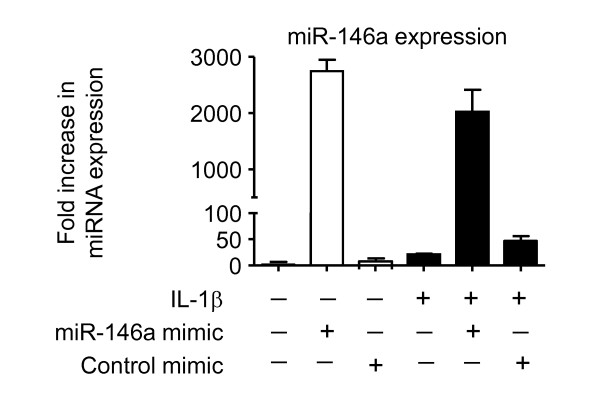
**Cellular miR-146a expression levels**. HASM cells were electroporated in the presence of buffer, control mimic or miR-146a mimic. Following repeated washes, cells were exposed to vehicle control or 1 ng/ml IL-1β and the levels miR-146a were measured by TaqMan RT-PCR at 24 h. The results are mean ± SEM of three independent experiments.

To confirm efficient transfection, the levels of miR-146a in cells electroporated with miR-146a mimics were measured by TaqMan and showed efficient transfection (Figure 7 - see below). Under the same condition, we have also demonstrated complete abolition of miR-146a expression in the presence of miR-146a inhibitor (data not shown). To provide additional evidence of transfection, we undertook parallel studies that examined the effect of an siRNA (100 nM) targeted to IL-6 and showed a 50% reduction in IL-6 release but no significant action upon IL-8 generation following IL-1β-stimulation (Figure 6C).

To understand the reason that miR-146a mimics reduced IL-1β-induced IL-6 and IL-8 release (Figure 6B), we measured the levels of miR-146a in HASM cells. These studies were performed following transfection with 100 nM miR-146a mimic since this concentration inhibited IL-1β-induced IL-6 and IL-8 release (Figure 6B). Significantly, cellular miR-146a levels were increased by 3000-fold following electroporation in the presence of miR-146a mimic, compared with the 20-50-fold increase in response to IL-1β exposure (Figure 7). This observation would suggest that although miR-146a mimics can attenuate IL-6 and IL-8 release, this is a false positive observation that is likely to be due to supra-maximal levels miR-146a levels which cannot be attained following exposure to IL-1β. Overall, the studies using JNK-1/2 and miR-146a inhibitors indicate that IL-1β-induced miR-146a expression is not central to the negative feedback regulation of IL-6 and IL-8 release.

### IL-1β-induced miR-146a expression does not regulate proliferation

**Figure 8:**
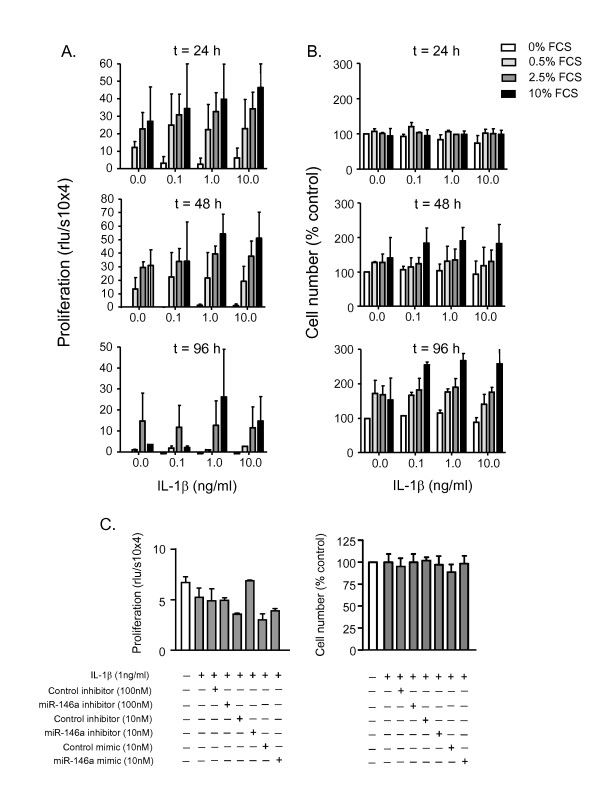
**Effect of miR-146a inhibitors and mimics upon IL-1β-induced proliferation and cell number**. HASM cells were exposed to either vehicle control, the indicated FCS concentration or the indicated concentration of IL-1β for 48 h, 72 h or 96 h before measurement of proliferation (A) or cell number (B). In panel C, HASM cells were electroporated in the presence of buffer, control inhibitor or miR-146a inhibitor, control mimic or miR-146a mimic. Cells were then exposed to vehicle control or 1 ng/ml IL-1β and proliferation and cell number was measured at 48 h. These results are mean ± SEM of three independent experiments.

Since previous studies have indicated that changes in miR-146a expression might regulate proliferation in a range of cancer cell lines [62,63] we therefore decided to investigate whether IL-1β-induced miR-146a expression might regulate HASM proliferation. Under the fetal calf serum (FCS)-free conditions used in these studies, IL-1β at concentrations up to 10 ng/ml did not induce a significant increase in HASM proliferation (Figure 8A) or cell number (Figure 8B) at 48 h, 72 h and 96 h. In contrast, FCS induced a concentration-dependent increase in proliferation at 48 h and 72 h (Figure 8A) which was reflected in an increase in cell number at 72 h and 96 h (Figure 8B). Given that IL-1β failed to impact upon proliferation and cell number, this suggested that miR-146a does not regulate these responses in HASM. To provide additional evidence to support this conclusion, we examined the role of miR-146a inhibitors and mimics at 48 h upon basal proliferation i.e. in the absence of FCS. From Figure 8C, it can be seen that neither miR-146a inhibitors or mimics had an effect upon basal proliferation or cell number in IL-1β-stimulated HASM cells.

### Mechanism of inhibition of IL-6 and IL-8 release by miR-146a mimics

**Figure 9:**
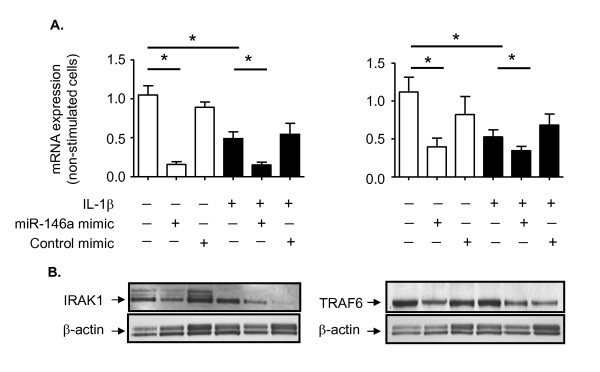
**IRAK-1 and TRAF6 mRNA and protein expression following exposure to IL-1β and miR-146a mimic**. HASM cells were transfection with either miR-146a mimics or control and then exposed to either vehicle control or 1 ng/ml IL-1β and the expression of IRAK-1 and TRAF6 mRNA (A) and protein (B) were detected at 24 h. The results in panel A are mean ± SEM of three independent experiments where * p < 0.05 versus time-matched controls whilst panel B is representative of three independent experiments.

Previous studies have indicated that inhibition of inflammatory mediator release by miR-146a is mediated through the down-regulation of IRAK-1 and TRAF6, which have multiple, predicted, miR-146a binding sites and form part of the common intracellular pathway that is activated via TLR/IL-1Rs [32,49]. Therefore, studies were undertaken to determine whether increased miR-146a levels following transfection with miR-146a mimics impacted on IRAK-1 and TRAF6 expression. Examination of IRAK-1 and TRAF6 mRNA expression showed a significant reduction of 51% and 55% at 24 h following IL-1β stimulation, respectively (Figure 9A). However, this reduction in mRNA expression was not reflected by a concomitant decrease in IRAK-1 and TRAF6 protein expression (Figure 9B). Exposure of non-stimulated cells to the miR-146a mimic resulted in a 84% and 62% reduction in the IRAK-1 and TRAF6 mRNA expression and further reductions in IRAK-1 and TRAF6 expression in IL-1β-stimulated HASM cells from 51% to 15% (IRAK-1) and 55% to 37% (TRAF6) (Figure 9A). Significantly, these reductions in IRAK-1 and TRAF6 mRNA levels were also reflected by a decrease in IRAK-1 and TRAF6 protein expression in both control and IL-1β-stimulated HASM cells in the presence of miR-146a mimic (Figure 9B). The control mimic had no effect upon IRAK-1 and TRAK6 mRNA expression (Figure 9A) but appeared to cause a non-selective reduction in IRAK-1 and TRAF6 protein expression in IL-1β treated but not control cells (Figure 9B). The reason for this reduction is unknown although we speculate that mimic controls might interact with pathways that regulated IRAK1 and TRAF6 translation but not transcription in activated cells.

**Figure 10:**
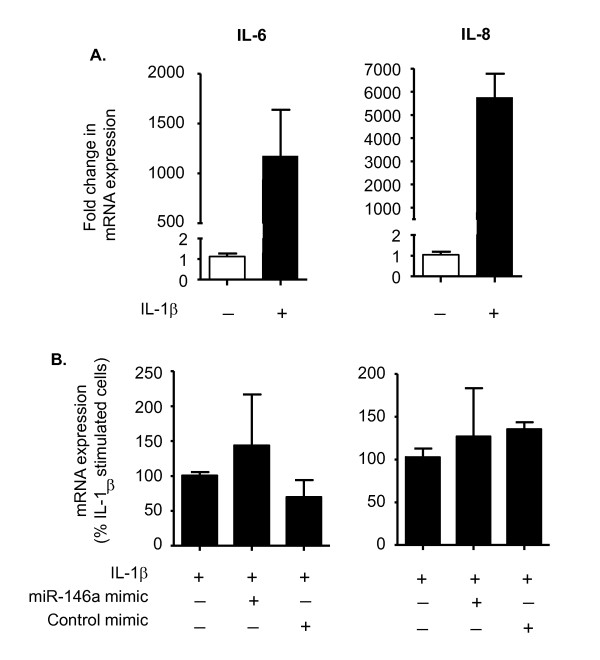
**Effect of miR-146a mimics upon IL-1β-induced IL-6 and IL-8 mRNA expression**. HASM cells were electroporated in the presence of buffer, control mimic or miR-146a mimic. Cells were then exposed to vehicle control or 1 ng/ml IL-1β and the expression of IL-6 and IL-8 mRNA was measured by qRT-PCR at 24 h. The results are the mean ± SEM of three independent experiments. Panel A shows the absolute increases in IL-6 and IL-8 mRNA whilst the results in panel B are expressed as a % compared to IL-1β stimulation (100%).

Since the miR-146a mimics reduced both IRAK-1 and TRAF6 mRNA and protein expression, we examined whether this could account for the inhibition of IL-6 and IL-8 release. To this end, we determined the effect of the miR-146a mimics on IL-1β induced IL-6 and IL-8 mRNA production. Exposure of HASM cells to IL-1β produced 1100- and 5700-fold increases in the levels of IL-6 and IL-8 mRNA, respectively (Figure 10A). Despite the fact that the miR-146a mimics had been previously shown to attenuate extracellular IL-6 and IL-8 release, we observed no significant inhibition of IL-6 or IL-8 mRNA expression (Figure 10B). These mechanistic studies indicate that although over-expression of miR-146a following transfection with miRNA mimics can partially down-regulate IRAK-1 and TRAF6 protein expression, this is not responsible for inhibition in IL-6 and IL-8 release from HASM. Instead, the action of the miR-146a mimics is mediated at a post-transcriptional stage following IL-6 and IL-8 synthesis.

## Discussion

Taganov *at al *[32] were the first to demonstrate increased miR-146a expression following activation of the TLR/IL-1R pathway. They also speculated that this might negatively regulate the innate immune response through down-regulation of IRAK-1 and TRAF6, two proteins that are involved in TLR/IL-1R signalling [32]. In the intervening period, the potential role of miR-146a as a negative regulator of the immune response has been highlighted by studies showing TLR/IL-1R-mediated miR-146a expression in multiple cell types and that changes in miR-146a expression is associated with inflammatory diseases including rheumatoid arthritis, osteoarthritis and systemic lupus erythematosus [32,48-54]. Surprisingly, only a few of these studies have demonstrated a functional link between miR-146a expression and the release of inflammatory mediators or have attempted to characterise the targets of miR-146a and its mechanism of action. In addition, despite the early demonstration that miR-146a expression is regulated at the transcriptional level through NF-κB activation, no reports have examined whether miR-146a production is also controlled at the post-transcriptional level [32,48,49,55,56]. For this reason, we have characterised the role of miR-146a during IL-1β-induced IL-6 and IL-8 release from primary HASM cells, which are known to contribute towards chronic inflammation associated with the development of asthma.

Initial studies demonstrated IL-1β-induced expression of miR-146a but not miR-155, miR-146b or miR-146*. Interestingly, a recent report by Kuhn *et al *[64] that examined the action of a combination of inflammatory mediators that included IL-1β, TNF-α and IFN-γ did not observe an increase in miR-146a expression. Instead, this study demonstrated down-regulation of multiple miRNAs and proceeded to show that reduced miR-25 expression increased the release of inflammatory mediators, extracellular matrix turnover and production of contractile proteins through up-regulation of Krüppel-like factor 4 (KLF4), a target of miR-25 [64].

Examination of the kinetics of miR-146a generation showed that this increased throughout the 72 h period following IL-1β stimulation although there appeared to be differences in the magnitude of the IL-1β-induced miR-146a expression, which we believe to be the result of patient-to-patient variation. Interestingly, these observations differed from previous studies in monocytes/macrophages and alveolar epithelial cells, where there was a rapid induction of miR-146a expression that peaked at 6-8 h [32,48,49]. We speculated that this prolonged miR-146a expression might impact upon other HASM functions such as differentiation or contractile potential. Indeed, studies in C2C12 skeletal muscle cell line have shown cyclic stretch induced miR-146a expression and that this promotes proliferation and inhibits differentiation through down-regulation of Numb, an inhibitor of Notch-induced differentiation [63]. Furthermore, a number of investigators have implicated changes in miR-146a expression in metastasis and proliferation associated with the development of papillary thyroid carcinoma (PTC) [65-67], cervical cancer [68], ovarian cancer [69], breast cancer [69-71], pancreatic cancer [70] and prostate cancer [70,72].

Having demonstrated IL-1β-induced miR-146a expression in HASM cells, we next investigated the mechanisms that regulate the transcription of primary miR-146a and its subsequent metabolism to produce the mature miR-146a. Previous studies in HASM cells have shown that exposure to IL-1β activates NF-κB (via IKK2) [6,16,17] and the MAP kinase pathways terminating at ERK-1/2, JNK-1/2 and p38 MAP kinase [6,10,17-21]. Therefore, established pharmacological inhibitors that had previously been shown to attenuate IKK2 and MAP kinase activity in HASM [6,10,17-21] were used to examine the role of these intracellular pathways. Significantly, these studies indicated that miR-146a was regulated at both the transcriptional and post-transcriptional level. As previously reported [32,48,49,55,56], we showed that initial transcription of primary miR-146a was mediated through activation of NF-κB. In addition, we have demonstrated that ERK-1/2 and JNK-1/2 but not the p38 MAP kinase pathways regulate the processing of primary miR-146a to produce mature miR-146a. We attempted to confirm these pharmacological observations by using siRNA-mediated knockdown of ERK-1/2 and JNK-1/2 but observed inhibition of IL-1β-induced miR-146a production in the presence of control siRNA. Dicer is thought to cleave the precursor miRNA to produce the double stranded miRNA and in combination with TRBP, is required for the loading of both siRNA and miRNAs into the Ago2 containing RISC complex. We therefore speculate that transfected siRNA might compete with precursor miR-146a for Dicer binding and by this route, siRNA could block the production of mature miR-146a. Significantly, competition between siRNA and miRNA has recently been demonstrated by Khan A *et al*. [73]. Overall, this is the first report demonstrating a role for ERK-1/2 and JNK-1/2 pathways in the regulation of miR-146a biogenesis and although the mechanism is presently unknown, we speculate that these MAP kinases might regulate proteins involved in miRNA processing or stability.

Examination of the effect of these MAP kinase inhibitors upon generation of inflammatory mediators showed that IL-6 release was mediated via NF-κB, ERK-1/2 and p38 MAP kinase whilst IL-8 release was mediated via NF-κB and ERK-1/2. Significantly, since neither IL-6 nor IL-8 release is influenced by the JNK-1/2 inhibitor, it was possible to use the JNK-1/2 inhibitor to examine the function of miR-146a during IL-1β-induced IL-6 and IL-8 release.

Previous investigations in alveolar epithelial cells, monocytes and macrophages have shown that increased levels of miR-146a negatively regulate the release of inflammatory mediators [32,48,49]. Transfection with miR-146a mimics, which caused a ~3000-fold increase in cellular miR-146a levels, could also inhibit IL-1β-induced IL-6 and IL-8 release in HASM cells. However, we showed that the ~100-fold increase in miR-146a expression following IL-1β stimulation is insufficient to inhibit IL-6 and IL-8, since attenuation of miR-146a activity (using a miR-146a inhibitor) or blocking miR-146a expression (using the JNK-1/2 inhibitor) had no significant effect upon cytokine release. It therefore appears that other mechanisms negatively regulate the release of these inflammatory mediators in HASM cells and that the inhibition in the presence of miR-146a mimic is a false positive observation resulting from the high cellular miR-146a levels [74,75].

Since IL-1β has also been shown to induce proliferation in ASM obtained from guinea-pig and rat trachea, we also decided to examine whether changes in miR-146a expression regulated this biological response [11-13]. However, we were unable to show increases in proliferation or cell number in human ASM following IL-1β exposure whilst miR-146a inhibitors and mimics had no effect upon the basal proliferation rate.

We next examined whether increases in miR-146a levels following IL-1β stimulation or transfection with miR-146a mimics could target down-regulation of IRAK-1 or TRAF6 protein expression as previously reported in monocytes/macrophages [32,49]. Interestingly, although we observed a reduction in IRAK-1 and TRAF6 mRNA expression following IL-1β exposure, this was not reflected in a reduction in protein levels. In contrast, miR-146a over-expression following transfection with miR-146a mimics caused a partial down-regulation in IRAK-1 and TRAF6 protein expression and a reduction in IL-6 and IL-8 secretion. However, as with our previous investigations in IL-1β-stimulated alveolar epithelial cells [48], the fact that miR-146a mimic failed to inhibit IL-1β-induced IL-6 and IL-8 mRNA production suggests that its action is mediated at a stage following IL-6 and IL-8 transcription and not through the down-regulation of TRAF6 and IRAK1. Although the mechanism of action is unknown, we speculated that the miR-146a mimic might down-regulate protein(s) involved in one or more steps including IL-6 and IL-8 translation and/or secretion.

## Conclusion

We have shown that IL-1β-induced a time- and concentration-dependent increase in miR-146a expression. As with miR-155 and the regulation of the immune response [30], we demonstrate that the function of miR-146a expression is cell-type specific. Thus, unlike alveolar epithelial cells and monocytes/macrophages, increased miR-146a expression following activation of the innate immune response does not appear to negatively regulate the release of inflammatory mediators in HASM cells. This may reflect the fact that the increases in miR-146a expression were insufficient to down-regulate the expression of IRAK-1, TRAF6 or other proteins that are involved in regulating the release of inflammatory mediators [32,48,49]. We have also shown that unlike ASM derived from guinea-pigs and rats, IL-1β does not induce proliferation in HASM and that IL-1β-induced miR-146a expression does not regulate basal proliferation in HASM. Interestingly, this study also demonstrates that the processing of primary miR-146a is regulated by the MAP kinases, ERK-1/2 and JNK-1/2. Given that activation of these MAP kinases has been demonstrated in a host of biological responses, it will be interesting to determine the mechanism by which MAP kinases regulate the biogenesis of miR-146a and other miRNAs.

## Competing interests

The authors declare that they have no competing interests.

## Authors' contributions

HML performed all the cell based studies and drafted the manuscript; ET and XJ measured the levels of primary miR-146a, AEW and MMP participated in the conception and design of the study: KFC and MAL participated in the conception and design of the study and were involved in drafting the manuscript. All authors have read and approved the final manuscript.

## Supplementary Material

Additional file 1 **Effect of inhibitors of IKK2 and MAP kinases upon HASM cell viability**. HASM cells were pre-treated for 60 min with the indicated concentrations of the inhibitors of IKK-2 (TPCA-1), MEK-1/2 (PD098059), JNK-1/2 (SP600125) and p38 MAP kinase (SB203580). Following exposure to IL-1β (1 ng/ml) for 24 h, cell viability was measured using an MTT assay. Results are expressed as the % of non-treated cells and are the mean ± SEM of 3 independent experiments. Click here for file
