# Supplementary figures and images for: Pharmacological studies of the mechanism and function of interleukin-1β-induced miRNA-146a expression in primary human airway smooth muscle
Source: Respir Res. 2010 Jun 2;11(1):68. doi: 10.1186/1465-9921-11-68 (PMC2894768; doi:10.1186/1465-9921-11-68)

## Slide 1
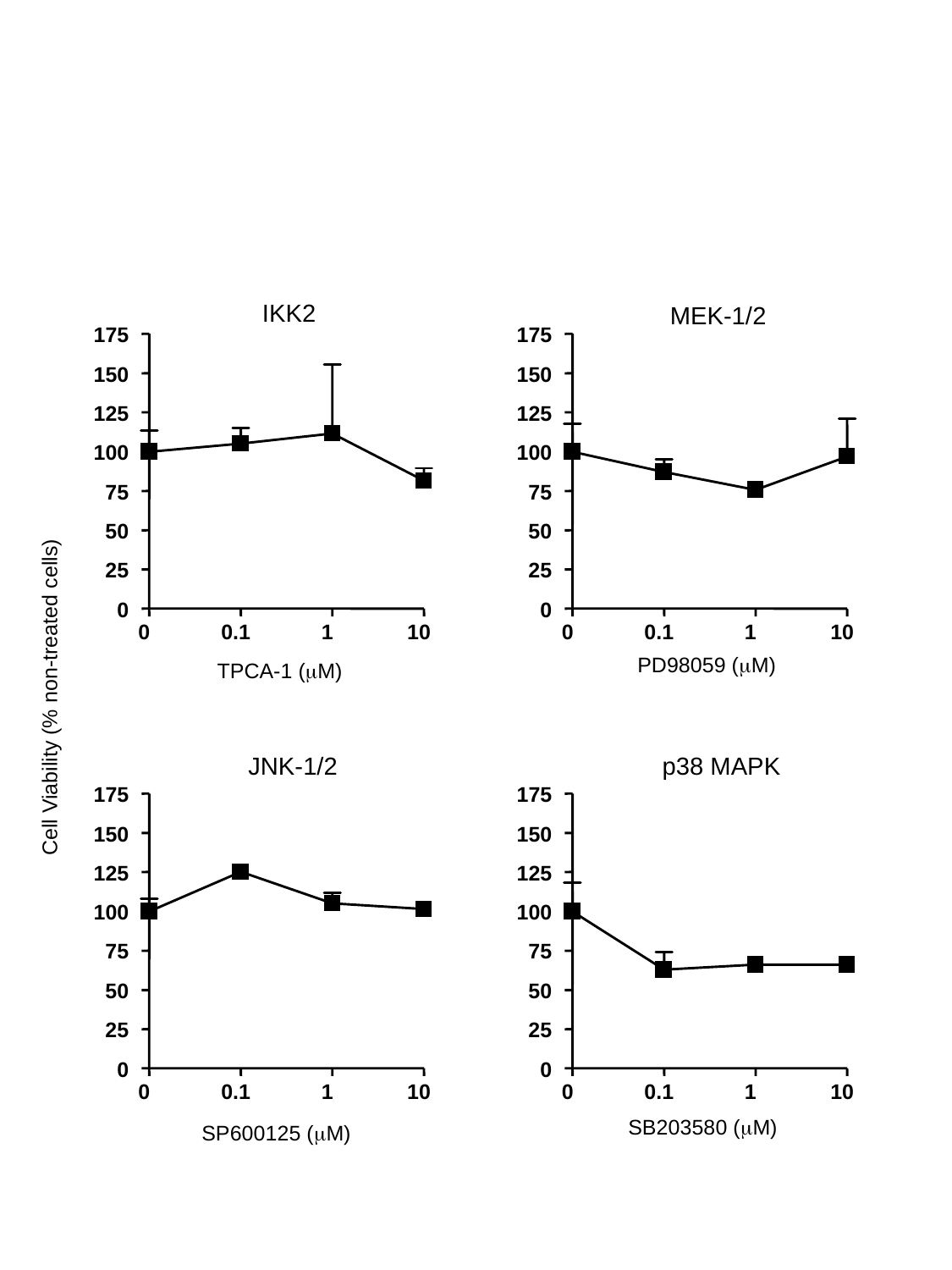

IKK2
MEK-1/2
175
175
150
150
125
125
100
100
75
75
50
50
25
25
0
0
0
0.1
1
10
0
0.1
1
10
PD98059 (μM)
TPCA-1 (μM)
Cell Viability (% non-treated cells)
JNK-1/2
p38 MAPK
175
175
150
150
125
125
100
100
75
75
50
50
25
25
0
0
0
0.1
1
10
0
0.1
1
10
SB203580 (μM)
SP600125 (μM)

Supplement: Additional file 1 — Effect of inhibitors of IKK2 and MAP kinases upon HASM cell viability. HASM cells were pre-treated for 60 min with the indicated concentrations of the inhibitors of IKK-2 (TPCA-1), MEK-1/2 (PD098059), JNK-1/2 (SP600125) and p38 MAP kinase (SB203580). Following exposure to IL-1β (1 ng/ml) for 24 h, cell viability was measured using an MTT assay. Results are expressed as the % of non-treated cells and are the mean ± SEM of 3 independent experiments. [file 1465-9921-11-68-S1.PPT]
